# Consistent safety and tolerability of Valtoco^®^ (diazepam nasal spray) in relationship to usage frequency in patients with seizure clusters: Interim results from a phase 3, long‐term, open‐label, repeat‐dose safety study

**DOI:** 10.1002/epi4.12494

**Published:** 2021-05-13

**Authors:** Ian Miller, James W. Wheless, Robert E. Hogan, Dennis Dlugos, Victor Biton, Gregory D. Cascino, Michael R. Sperling, Kore Liow, Blanca Vazquez, Eric B. Segal, Daniel Tarquinio, Weldon Mauney, Jay Desai, Adrian L. Rabinowicz, Enrique Carrazana, The DIAZ.001.05 Study Group

**Affiliations:** ^1^ Formerly Nicklaus Children's Hospital Miami FL USA; ^2^ Le Bonheur Children's Hospital University of Tennessee Health Science Center Memphis TN USA; ^3^ Washington University in St. Louis St. Louis MO USA; ^4^ Children's Hospital of Philadelphia Philadelphia PA USA; ^5^ Arkansas Epilepsy Program Little Rock AR USA; ^6^ Mayo Clinic Rochester MN USA; ^7^ Thomas Jefferson University Philadelphia PA USA; ^8^ Hawaii Pacific Neuroscience Honolulu HI USA; ^9^ Comprehensive Epilepsy Center New York University New York NY USA; ^10^ Hackensack University Medical Center and Northeast Regional Epilepsy Group Hackensack NJ USA; ^11^ Center for Rare Neurological Diseases Atlanta GA USA; ^12^ Northwest Florida Clinical Research Group Gulf Breeze FL USA; ^13^ Children's Hospital of Los Angeles Los Angeles CA USA; ^14^ Neurelis, Inc. San Diego CA USA

**Keywords:** acute repetitive seizure, diazepam, dosing frequency, intranasal, nasal spray, seizure cluster

## Abstract

**Objective:**

Need for rescue therapy differs among patients with seizure clusters. Diazepam nasal spray is approved to treat seizure clusters in patients with epilepsy ≥6 years of age. This analysis used interim data from a phase 3 safety study to assess safety profile and effectiveness of diazepam nasal spray using average number of doses/month as a proxy measurement.

**Methods:**

This phase 3, open‐label, repeat‐dose, safety study of diazepam nasal spray enrolled patients (6‐65 years) with epilepsy and need of benzodiazepine rescue. Patients were stratified by average number of doses/month (<2, moderate frequency; 2‐5, high frequency; >5, very‐high frequency). Safety was evaluated based on treatment‐emergent adverse events (TEAEs), assessed nasal irritation, and olfaction. The proportion of treatments given as a second dose was used as an exploratory proxy for effectiveness.

**Results:**

Of 175 enrolled patients (data cutoff, October 31, 2019), 158 received ≥1 dose of diazepam nasal spray. Frequency of use was moderate in 43.7% of patients, high in 50.6% of patients, and very high in 5.7% of patients. Patients treated 3397 seizure episodes (moderate frequency, 14.2%; high frequency, 59.9%; very high frequency, 25.8%). Nasal discomfort was the most common treatment‐related TEAE in all groups. No notable changes in nasal irritation or olfaction were observed. Second doses represented only 2.5%, 7.5%, and 17.2% of all doses in the moderate‐, high‐, and very‐high‐frequency groups, respectively. Overall retention rate was 82.9%, without an observed relationship to frequency of use.

**Significance:**

Frequency of dosing diazepam nasal spray had little impact on the safety/tolerability profile across a range of <2 to >5 doses/month. Effectiveness was suggested for all dosing frequencies by the high proportion of seizure clusters not treated with a second dose. These results support the utility, safety profile, and effectiveness of diazepam nasal spray across frequencies of seizure cluster burden.

**Key Points**

- Diazepam nasal spray is indicated for the treatment of seizure clusters in patients with epilepsy age ≥6 years
- This phase 3 safety study of diazepam nasal spray included 158 patients, treating 3397 seizure episodes over a median of 396 days
- In this interim analysis, patient subgroups were analyzed by average monthly dosing of diazepam nasal spray: <2, 2‐5, or >5 doses
- Frequency of dosing diazepam nasal spray had little impact on the safety/tolerability profile across a range of <2 to >5 doses/month
- The proportion of second doses used across dosing frequencies (9.3%) was low, and the retention rate (82.9%) was high

## 1 INTRODUCTION

Epilepsy treatment relies on a stable regimen of antiseizure medications (ASMs); however, some treated patients may still experience seizure clusters (also called acute repetitive seizures).^1^ These seizure emergencies are associated with an increased risk of progression to status epilepticus,^2^ more frequent emergency room visits, and disruption of the daily life and work of both patients and care partners.^3^ Treatment of seizure clusters with rescue medications may reduce the need for emergency care.^4^

Benzodiazepines are most commonly used as rescue therapy. Rectal diazepam (Diastat^®^)^5^ has been used for the treatment of seizure clusters for more than 20 years and is suitable for administration by non–healthcare professionals. Rectal diazepam may be difficult to administer during an active seizure and has wide pharmacokinetic patient‐to‐patient variability.^6^, ^7^ In practice, buccal and oral benzodiazepines have also be used as rescue therapy for seizure clusters^1^; however, these are not approved for this use in the United States. Intranasal treatment is designed to overcome the drawbacks of rectal administration, with potential advantages such as absorption by the nasal mucosa and bypassing the gastrointestinal tract and hepatic first‐pass effects, in addition to being more accessible during a seizure and less potentially embarrassing for patients and care partners than rectal administration.^7^, ^8^

Diazepam nasal spray (Valtoco^®^) is indicated for the acute treatment of intermittent, stereotypic episodes of frequent seizure activity that are distinct from a patient's usual seizure pattern in patients with epilepsy 6 years of age and older; episodes of frequent seizure activity can include seizure clusters or acute repetitive seizures. In a study in 48 healthy adults, diazepam nasal spray provided a rapid and noninvasive route of administration and had comparable bioavailability to rectal diazepam overall, but with less intrapatient variability.^9^ The safety profile was consistent with that expected for rectal diazepam.^9^, ^10^

Occurrence of seizure clusters, and therefore usage frequency of rescue therapy, varies among patients, so it is important to determine whether long‐term safety is related to monthly usage frequency. In the present study of diazepam nasal spray, we investigated this using interim data from a phase 3 study of patients with epilepsy who treated moderately frequent, frequent, or very frequent seizure clusters over 12 months or more. Here, we present the safety, tolerability, and effectiveness (with use of a second dose as a proxy) results stratified according to the average number of doses per month.

## 2 METHODS

### 2.1 Study design

This phase 3, open‐label, repeat‐dose safety study of diazepam nasal spray was conducted in 24 centers in the United States (ClinicalTrials.gov identifier: NCT02721069). The study received approval from each site's institutional review board and was conducted in accordance with the Declaration of Helsinki. Written informed consent was provided by all patients or their parents/guardians prior to study participation. The primary endpoint of this study is assessment of safety of diazepam nasal spray. This interim analysis (data cutoff as of October 31, 2019) also evaluated whether seizure episodes were treated with a second dose in the same calendar day (as a proxy for effectiveness) stratified by frequency of use based on the average number of doses/month (<2 doses/month [moderate usage frequency], 2‐5 doses/month [high usage frequency], and >5 doses/month [very‐high usage frequency]).

The study design comprised a screening phase, baseline, and a 12‐month treatment period, after which patients could elect to continue therapy. Doses of diazepam nasal spray were based on the patient's age and body weight. Patients and care partners were trained on the proper use of the nasal sprayer and were instructed that a second dose could be administered, if needed, 4‐12 hours after the first dose and to allow 5 days before repeating the dose. Investigators could adjust doses for safety or efficacy if there was no safety concern. A patient diary was used to record seizures and drug administration.

### 2.2 Patients

Patients were males and females aged 6‐65 years (inclusive), with a clinical diagnosis of epilepsy (either partial or generalized epilepsy with motor seizures or seizures with clear alteration of awareness) and frequent seizure clusters (ie, acute repetitive seizures with bouts of uncontrolled seizures), who were on a stable regimen of ASMs but, in the investigator's opinion, might need benzodiazepine intervention for seizure control on average once every other month (mean of six times/year). Patients had to have a qualified care partner or medical professional available who could administer study medication in the event of a seizure. Female patients of childbearing potential agreed to use an approved method of birth control. Patients also were required to have no clinically significant abnormal findings in their medical history or on physical examination, electrocardiogram (Fridericia's correction formula <450 msec for males and <470 msec for females), or clinical laboratory results during screening. Other key exclusion criteria were a history of major depression, a past suicide attempt, suicide ideation, a history of allergy or adverse response to diazepam, or a history of a clinically significant medical condition that would jeopardize the safety of the patient. Previous status epilepticus, concomitant use of other benzodiazepines, and history of seasonal allergies were not exclusion criteria.

### 2.3 Safety

Safety was evaluated based on incidence of treatment‐emergent adverse events (TEAEs), reported as preferred terms from the *Medical Dictionary for Regulatory Authorities,* and physical/neurological examination. Tolerability assessments included olfactory changes on the NIH Toolbox Odor Identification Test,^11^ in which participants used scratch‐and‐sniff cards and, after scratching them one at a time, were asked to identify which of four pictures on a tablet screen matches the odor. Scores were calculated by summing the total number of correct items, with higher scores indicating better olfaction. Nasal irritation was measured objectively by a trained observer on a 6‐point scale, with grade 0 indicating no sign of nasal irritation or mucosal erosion, 1A–focal nasal mucosal irritation or inflammation, 1B–superficial mucosal erosion, 2–moderate mucosal erosion, 3–ulceration, and 4–septal perforation.^12^

### 2.4 Effectiveness

The proportion of seizure events that were treated with a second dose of diazepam nasal spray (ie, both an initial and a second dose) in a calendar day was used as an exploratory measure and proxy for effectiveness; there were no prespecified efficacy endpoints.

## 3 RESULTS

### 3.1 Study population

A total of 175 patients were enrolled at the October 31, 2019, interim cutoff date; 158 were treated with diazepam nasal spray and included in the safety population. The demographic and baseline characteristics are shown in Table 1. Frequency of use of diazepam nasal spray was moderate (<2 doses/month) in 69 (43.7%) patients, high (2‐5 doses/month) in 80 (50.6%) patients, and very high (>5 doses/month) in 9 (5.7%) patients (Figure S1).

**TABLE 1:** Demographic and baseline characteristics of the interim data population

Variable	Total (n = 158)	Moderate‐frequency usage (<2 doses/month) (n = 69)	High‐frequency usage (2‐5 doses/month) (n = 80)	Very‐high‐frequency usage (>5 doses/month) (n = 9)
Age, y, mean (SD)	23.5 (15.1)	22.1 (13.8)	25.3 (16.6)	18.1 (9.2)
Min, max	6, 65	6, 58	6, 65	6, 35
Median	19.5	19.0	21.0	15.0
Sex, n (%)				
Male	73 (46.2)	33 (47.8)	39 (48.8)	1 (11.1)
Female	85 (53.8)	36 (52.2)	41 (51.3)	8 (88.9)
Race, n (%)				
White	130 (82.3)	57 (82.6)	65 (81.3)	8 (88.9)
Black/African American	15 (9.5)	5 (7.2)	9 (11.3)	1 (11.1)
Asian	4 (2.5)	3 (4.3)	1 (1.3)	0
Native Hawaiian/Pacific Islanders	5 (3.2)	2 (2.9)	3 (3.8)	0
Other	4 (2.5)	2 (2.9)	2 (2.5)	0
Height, cm, mean (SD)	152.7 (23.2)	152.0 (25.4)	153.9 (21.4)^a^	147.9 (23.2)
Weight, kg, mean (SD)	60.9 (33.7)	58.9 (32.1)	63.0 (35.6)	58.1 (30.5)

Abbreviation: SD, standard deviation.

^a^ n = 79.

**FIGURE 1:**
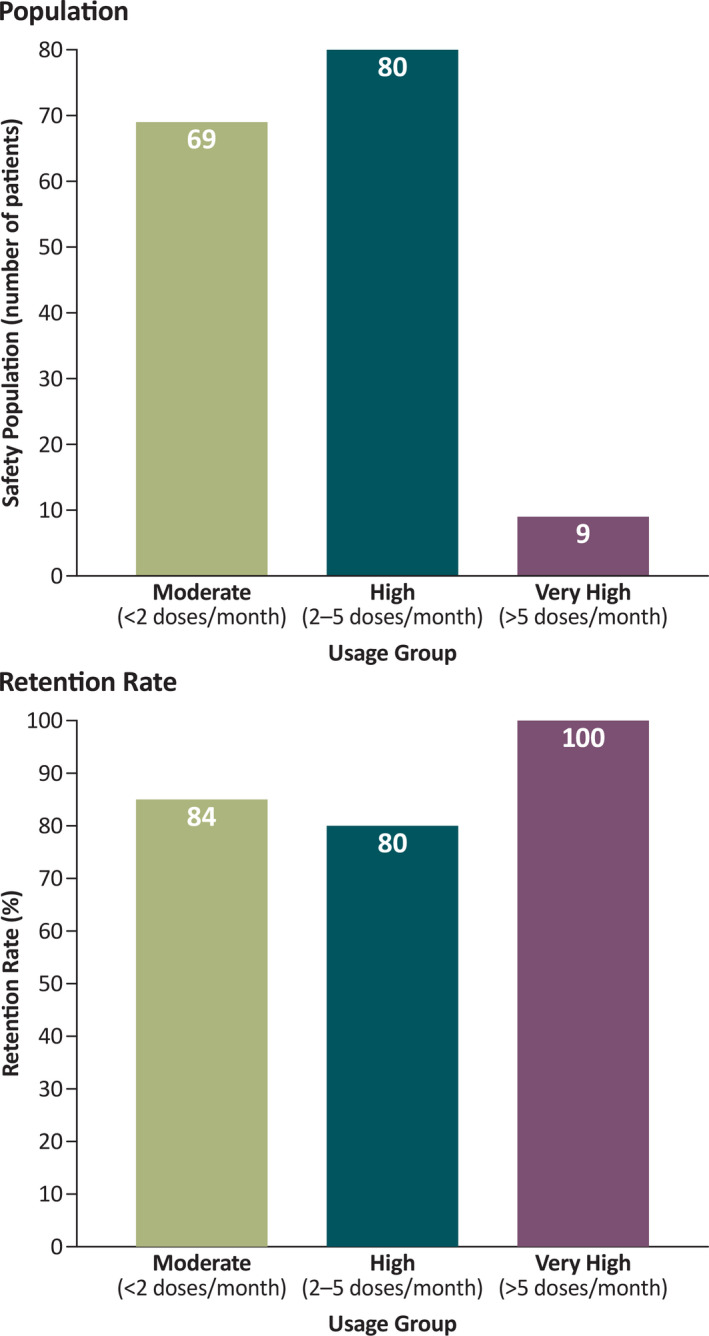
Population and retention rates for the usage groups

Among treated patients at the interim data cutoff point, there were 27 discontinuations, to give an overall retention rate of 82.9%. The retention rates for the moderate‐, high‐, and very‐high‐frequency usage groups were 84.0%, 80.0%, and 100%, respectively (Figure 1). At time of analysis, 47 patients (29.7% of the safety population) had completed the study and 84 patients (53.2%) were continuing treatment. There were no discontinuations due to adverse events (AEs).

#### 3.1.1 Exposure

The majority of patients (73.4%) were administering diazepam nasal spray for 12 months or more at the interim cutoff date (Table 2), with an overall median duration in the study of 396 days. During this time, patients treated 3397 seizure episodes, with 14.2% occurring in the moderate‐frequency group, 59.9% in the high‐frequency group, and 25.8% in the very‐high‐frequency group. Of note, although the very‐high‐frequency group represented only 5.7% of the study population, these patients recorded more than one‐fourth of all treated seizure episodes in the study.

**TABLE 2:** Diazepam nasal spray exposure and treated seizures at interim data cutoff

Variable	Total (n = 158)	Moderate‐frequency usage (<2 doses/month) (n = 69)	High‐frequency usage (2‐5 doses/month) usage (n = 80)	Very‐high‐frequency usage (>5 doses/month) (n = 9)
Duration of exposure, months, patients (%)				
<6 months	11 (7.0)	6 (8.7)	5 (6.3)	0
6 to <12 months	31 (19.6)	10 (14.5)	19 (23.8)	2 (22.2)
≥12 months	116 (73.4)	53 (76.8)	56 (70.0)	7 (77.8)
Seizure cluster episodes				
Treated seizure episodes, n	3397	484	2036	877
Second doses used to treat seizure episodes, n (%)	316 (9.3)	12 (2.5)	153 (7.5)	151 (17.2)

### 3.2 Safety

In the overall study population, 119 (75.3%) patients experienced a TEAE, irrespective of causality, and 45 (37.8%) of these were classified as serious (Table 3). Seizure and upper respiratory tract infections were the most common TEAEs in the moderate‐frequency group; nasopharyngitis and influenza were the most common in the high‐frequency group; and headache and nasal discomfort were the most common in the very‐high‐frequency groups (Table 4). Status epilepticus only occurred in the high‐frequency group. The only TEAEs occurring more than once in the very‐high‐frequency group were headache and nasal discomfort, with two events each (22.2%). The overall incidence of somnolence was seven events (4.4%). Cardiorespiratory events, which may be associated with high‐dose intravenous benzodiazepines,^13^, ^14^ including respiratory depression, hypotension, and arrhythmia, were not observed.

**TABLE 3:** Summary of treatment‐emergent adverse events

Variable, n (%)	Total (n = 158)	Moderate‐frequency usage (<2 doses/month) (n = 69)	High‐frequency usage (2‐5 doses/month) (n = 80)	Very‐high‐frequency usage (>5 doses/month) (n = 9)
Patients with TEAEs	119 (75.3)	49 (71.0)	63 (78.8)	7 (77.8)
Patients with serious TEAEs	45 (28.5)	22 (31.9)	20 (25.0)	3 (33.3)
Important medical events	8 (5.1)	4 (5.8)	3 (3.8)	1 (11.1)
Required/prolonged hospitalization	40 (25.3)	19 (27.5)	19 (23.8)	2 (22.2)
Patients with treatment‐related TEAEs^a^	26 (16.5)	8 (11.6)	15 (18.8)	3 (33.3)

Abbreviations: AE, adverse event; TEAE, treatment‐emergent adverse event.

^a^ Possibly or probably related to study drug.

**TABLE 4:** Treatment‐emergent adverse events ≥5% and ≥2 patients in any frequency of use group

TEAE, n (%)	Moderate‐frequency usage (<2 doses/month) (n = 69)	High‐frequency usage (2‐5 doses/month) (n = 80)	Very‐high‐frequency usage (>5 doses/month) (n = 9)^a^
Nasopharyngitis	1 (1.4)	10 (12.5)	1 (11.1)
Seizure	13 (18.8)	9 (11.3)	1 (11.1)
Influenza	1 (1.4)	6 (7.5)	1 (11.1)
Pyrexia	4 (5.8)	6 (7.5)	—
Somnolence	2 (2.9)	5 (6.3)	—
Status epilepticus	—	5 (6.3)	—
Upper respiratory tract infection	8 (11.6)	4 (5.0)	—
Pneumonia	6 (8.7)	4 (5.0)	1 (11.1)
Urinary tract infection	3 (4.3)	4 (5.0)	—
Nasal discomfort	3 (4.3)	4 (5.0)	2 (22.2)
Vomiting	3 (4.3)	4 (5.0)	—
Contusion	—	4 (5.0)	—
Headache	2 (2.9)	3 (3.8)	2 (22.2)
Dizziness	5 (7.2)	2 (2.5)	—
Sinusitis	4 (5.8)	2 (2.5)	—

Abbreviation: TEAE, treatment‐emergent adverse event.

^a^ In the >5 doses/month group, headache and nasal discomfort occurred in two patients, with no other TEAEs occurring in more than one patient.

The proportions of patients with TEAEs that were considered by the investigator to be possibly or probably related to the study drug were 11.6% and 18.8% in the moderate‐ and high‐frequency groups, respectively, and 33.3% in the small very‐high‐frequency group. The treatment‐related TEAEs occurring in more than one patient per group were as follows: nasal discomfort (3 [4.3%] patients) in the moderate group; nasal discomfort (4 [5.0%] patients), epistaxis (2 [2.5%] patients), rhinalgia (2 [2.5%] patients), headache (2 [2.5%] patients), and somnolence (2 [2.5%] patients) in the high‐frequency group; and nasal discomfort (2 [22.2%] patients) in the very‐high‐frequency group. The nasal discomfort occurred only in patients taking 15‐ and 20 ‐mg doses (moderate group, 2 and 1; high‐frequency group, 2 and 2; and very‐high‐frequency group, 0 and 2, respectively), and was typically mild and was not noted on subsequent visits.

The proportion of patients with serious TEAEs were 31.9% in the moderate‐, 25.0% in the high‐, and 33.3% in the very‐high‐frequency group (Table 3). The serious TEAEs occurring more than once are shown in Table 5; no serious TEAEs were considered by the investigator to be possibly or probably related to diazepam nasal spray treatment.

**TABLE 5:** Serious treatment‐emergent adverse events (>1 in any group)

Serious TEAE, n (%)	Moderate‐frequency usage (<2 doses/month) (n = 69)	High‐frequency usage (2‐5 doses/month) (n = 80)	Very‐high‐frequency usage (>5 doses/month) (n = 9)
Seizure	10 (14.5)	7 (8.8)	1 (11.1)
Seizure cluster	2 (2.9)	—	—
Status epilepticus	—	5 (6.3)	—
Pneumonia	4 (5.8)	3 (3.8)	—
Pyrexia	—	2 (2.5)	—

Abbreviation: TEAE, treatment‐emergent adverse event.

### 3.3 Nasal irritation and olfaction

The nasal irritation test showed that most patients experienced no sign of nasal or mucosal irritation. Cases of irritation were not noted on subsequent visits. In the high‐frequency group, 12 patients were recorded with mild nasal irritation (grade 1A) over the 30‐day to 365‐day period compared with none in the moderate‐frequency group. In the very‐high‐frequency group, only one patient recorded any degree of nasal irritation (grade 1A, on day 30) (Table S1). One patient in the high‐frequency group showed superficial mucosal erosion (grade 1B) at day 150 and one at day 330.

Smell tests using the NIH Toolbox Odor Identification test showed numerically small olfactory changes; however, clinically meaningful thresholds have not been identified for this subscale (NIH Toolbox Scoring and Interpretation Guide 2016). At measured timepoints from baseline to day 365 (Table S2), median change in scores from baseline ranged from −0.13 to 0.25 for the moderate‐frequency group, −0.47 to 0.89 for the high‐frequency group, and 0.5 to 1.5 for the very‐high‐frequency group; higher scores indicate better olfaction.

### 3.4 Effectiveness

The majority of seizure episodes were treated with a single dose of diazepam nasal spray within the same calendar day. In the moderate‐frequency group, 2.5% of doses were second doses, with 7.5% in the high‐frequency group and 17.2% in the very‐high‐frequency group (Table 2).

## 4 DISCUSSION

Rapid and effective treatment of seizure clusters is particularly important as they are associated with status epilepticus, which in one study occurred in 44% of patients with seizure clusters and 12.5% in patients without.^2^ Diazepam nasal spray addresses this need by providing rapid absorption, high bioavailability, and a good safety profile with a socially acceptable route of administration that is designed to be easy to use by nonmedical care partners. In this interim analysis of 158 patients with 3397 treated seizure episodes despite stable ASM regimens, diazepam nasal spray demonstrated a generally similar long‐term safety and tolerability profile across subgroups of patients with moderate (<2 doses/month), high (2‐5 doses/month), and very high (>5 doses/month) usage of diazepam nasal spray, and was consistent with diazepam's established safety profile. No new safety concerns were identified, and no trends were observed for TEAEs, nasal irritation, or olfactory changes with higher usage frequency. Exposure was at least 12 months in 73.4% of patients, and the retention rate was high at 82.9% overall, with 100% retention in the small (n = 9) very‐high‐frequency usage group.

The safety and tolerability profile for diazepam nasal spray was similar to that observed in previous studies.^9^, ^10^, ^15^ In this study, the most common TEAEs overall were seizure (14.6%), upper respiratory tract infection (7.6%), and nasopharyngitis (7.6%); none of which were considered to be related to diazepam nasal spray. Somnolence TEAEs were low at 4.4%, with none recorded in the very‐high‐frequency usage group. Treatment‐related TEAEs occurred in only 16.5% of patients overall, primarily nasal discomfort, which was generally mild and was not noted on subsequent visits. None of the TEAEs graded as serious were considered to be related to treatment. The incidence and types of TEAEs were similar across the three dose frequency groups, which is striking in light of the substantial seizure burden in the small, very‐high‐frequency usage group.

Nasal irritation, as assessed by a trained observer, was mild and was not noted on subsequent visits, with only a small proportion (1.3% to 3.8%) of high‐frequency patients experiencing nasal mucosal irritation, a level that was lower than at baseline, before patients had used diazepam nasal spray (4 [5.0%] patients with grade 1A irritation). In the very‐high‐frequency patients, there was only one case of grade 1A irritation, suggesting there is little concern that higher doses lead to clinically significant nasal irritation. There were no clinically significant changes in olfaction, and the minor changes observed were not associated with frequency of dosing.

Several proxy measures of effectiveness were analyzed in this study. Need for a second dose during a calendar day is an important parameter, because seizure clusters may persist for 24 hours.^16^ In a study of online diaries from 1177 patients recording two or more seizures in 24 hours, 58.2% of patients had a second seizure 6‐24 hours after the first. In the present study, a high percentage of seizure episodes were treated with only a single dose of diazepam nasal spray in a calendar day, with only 9.3% of doses being second doses. In addition, high retention rates in all groups (82.9% overall after a median 13 months of treatment) suggest that patients and their care partners found treatment to be beneficial, so continued to use the medication. Taken together, these data are supportive of real‐world effectiveness across a broad range of seizure burden and usage patterns.

Although patients and their care partners were instructed not to use diazepam nasal spray more than five times per month (consistent with current labeling), a small subgroup of patients (n = 9) in this open‐label, single‐arm, real‐world study did average more than five doses per month. The guidance on monthly dosing is consistent with that of diazepam rectal gel^5^; however, this timeframe may be conservative, and, to our knowledge, there are no data in patients with seizure clusters supporting a specific frequency limit. Given the context of the extensive, long‐term exposure to diazepam nasal spray and the high seizure burden of these patients, the results presented appear to be reassuring.

Due to the study's open‐label, uncontrolled design evaluating the safety of diazepam nasal spray, it was not powered for assessing statistical differences. Lack of statistical analysis may be considered a limitation of this study. Notwithstanding the descriptive nature of the analysis, the data presented here can be of practical value to prescribers.

In conclusion, the frequency of dosing diazepam nasal spray had little impact on the safety profile across a range of <2 to >5 doses/month, based on the interim data from this long‐term study. Cardiorespiratory events, which may be associated with high‐dose intravenous benzodiazepines, were not observed. Effectiveness was suggested for all dosing frequencies, as shown by the low number of seizure episodes treated with a second dose (and, therefore, the high number treated with a single dose) of diazepam nasal spray and low discontinuation rates. Together, these results support the utility and safety profile of diazepam nasal spray across frequency of seizure cluster burden.

## CONFLICT OF INTEREST

**Dr Miller** is currently an employee of Marinus Pharmaceuticals and has served as a consultant/advisor to GW Pharmaceuticals; Insys Therapeutics; Visualase; NeuroPace; and Neurelis, Inc; and as a study investigator for GW Pharmaceuticals. **Dr Wheless** has served as an advisor or consultant for: CombiMatrix; Eisai Inc; GW Pharmaceuticals; Lundbeck, Inc; Neurelis, Inc; NeuroPace, Inc; Supernus Pharmaceuticals, Inc; and Upsher‐Smith Laboratories, Inc Dr Wheless has served as a speaker or a member of a speakers bureau for: Cyberonics, Inc; Eisai Inc; Lundbeck, Inc; Mallinckrodt; Neurelis, Inc; Supernus Pharmaceuticals, Inc; Upsher‐Smith Laboratories, Inc, and has received grants for clinical research from: Acorda Therapeutics; GW Pharmaceuticals; Insys Therapeutics, Inc; Lundbeck, Inc; Mallinckrodt; Neurelis, Inc; NeuroPace, Inc; Upsher‐Smith Laboratories, Inc; and Zogenix, Inc **Dr Hogan** has received research support from UCB Pharmaceuticals, Neurelis, Inc; and Biogen Inc, and is an advisor for Neurelis, Inc **Dr Dlugos** receives salary support from NIH, Commonwealth of Pennsylvania Department of Health, Pediatric Epilepsy Research Foundation, and The Epilepsy Study Consortium. He is an investigator on research grants awarded to CHOP from Zogenix, Greenwich Biosciences, UCB, Brain Sentinel, Neurelis, Q‐State, USL, Aquestive, Bio‐Pharm, Insys, SK Life Sciences, and Encoded Therapeutics. He has received travel expenses for protocol development conferences or investigator meetings from Marinus, Ovid/Takeda, Ultragenyx, USL, Pfizer, and Zogenix. He received honoraria and/or travel support for CME and other educational programs from Wake Forest University School of Medicine, American Epilepsy Society, American Academy of Neurology, Epilepsy Foundation of America, Epilepsy Foundation of North Carolina, Medscape, Miller Medical Communications, Ecuador Neurology Project, Ministry of Health of the United Arab Emirates, and Seoul National University. **Dr Biton** has nothing to disclose. **Dr Cascino** has nothing to disclose. **Dr Sperling** has received personal compensation for speaking from NeurologyLive and Eisai, is an advisor for Neurelis, Inc, and consulting with payments to Thomas Jefferson University from Medtronic. Dr Sperling has received research support from Eisai; Medtronic; Neurelis, Inc; SK Life Science; Takeda; Sunovion; UCB Pharma; Xenon; and Engage Pharmaceuticals. **Dr Liow** has received research support from Intracellular Therapies, SK Life Sciences, Genentech, Biotie Therapies, MonoSol, Aquestive Therapeutics, Engage Therapeutics, Xenon, Lundbeck, Biogen, Eli Lilly, Pfizer, Novartis, Sunovion, Acorda, Eisai, UCB, LivaNova, Axsome, and Acadia. **Dr Vazquez** is an advisor for Neurelis, Inc **Dr Ayala** has nothing to disclose. **Dr Segal** has received personal compensation for consulting, serving on a scientific advisory board, speaking, or other activities with Eisai, Lundbeck, Nutricia, Novartis, Greenwich, Epitel, Encoded Therapeutics and QBioMed, and is an advisor for Neurelis, Inc **Dr Tarquinio** has received personal compensation for consulting, serving on a scientific advisory board, speaking, or other activities with Marinus, Avexis, and Neurelis, Inc **Dr Mauney** has nothing to disclose. **Dr Desai** has received research funding from the Epilepsy Foundation of Greater Los Angeles; Neurelis, Inc; Novartis; Ovid; Aquestive; and UCB. **Dr Rabinowicz** is an employee and has received stock options from Neurelis. **Dr Carrazana** is an employee of and has received stock and stock options from Neurelis, Inc Dr Carrazana has received compensation for serving on the boards of directors of Marinus and Hawaii‐Biotech. We confirm that we have read the Journal's position on issues involved in ethical publication and affirm that this report is consistent with those guidelines.

## Supporting information

Figure S1

Table S1‐S2
