# Supplementary figures and images for: Consistent safety and tolerability of Valtoco® (diazepam nasal spray) in relationship to usage frequency in patients with seizure clusters: Interim results from a phase 3, long‐term, open‐label, repeat‐dose safety study
Source: Epilepsia Open. 2021 May 13;6(3):504–12. doi: 10.1002/epi4.12494 (PMC8408590; doi:10.1002/epi4.12494)

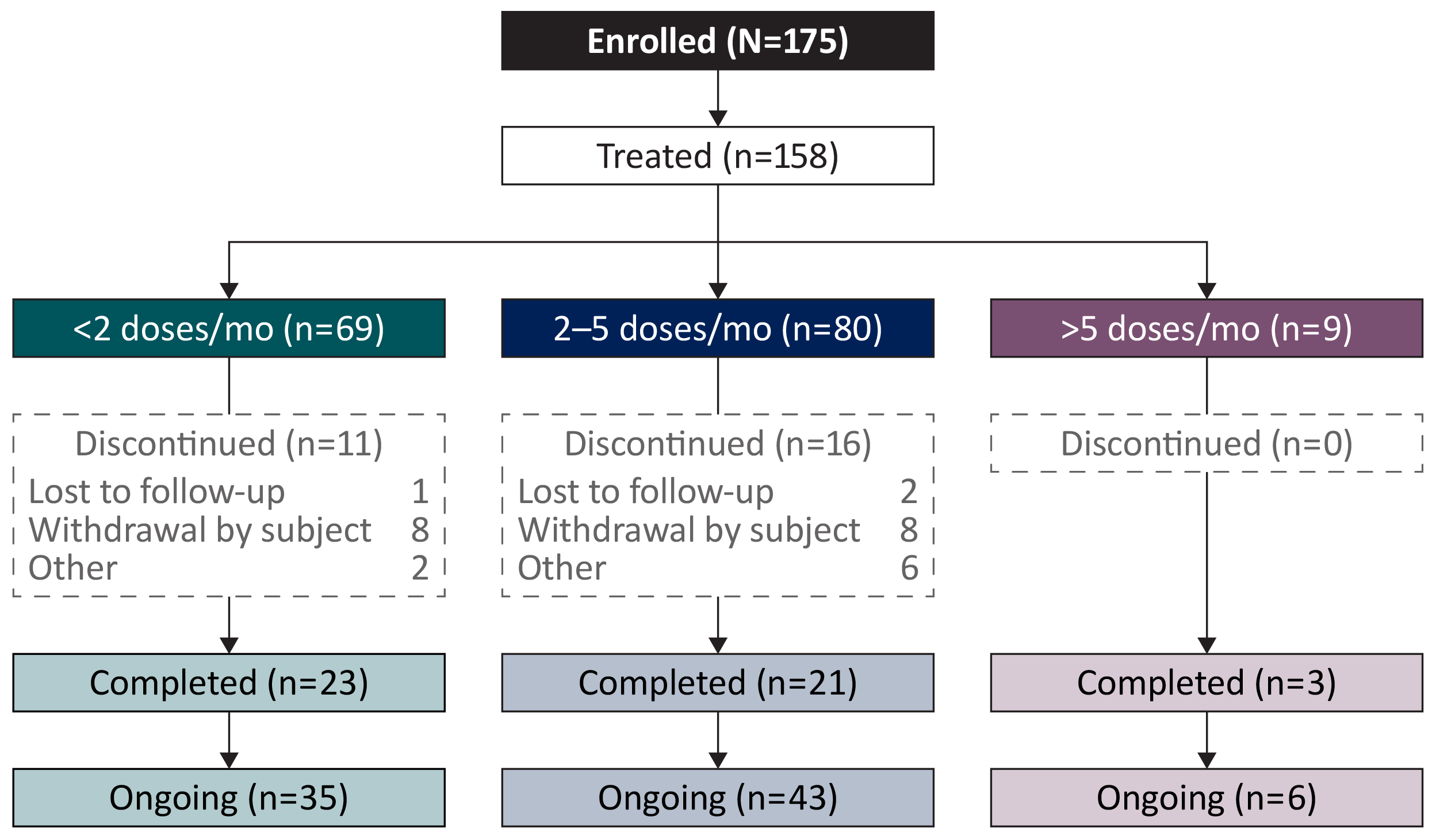

Supplement: Supplementary file 1 — Figure S1 [file EPI4-6-504-s001.tif]
