# Supplementary material for: Consistent safety and tolerability of Valtoco® (diazepam nasal spray) in relationship to usage frequency in patients with seizure clusters: Interim results from a phase 3, long‐term, open‐label, repeat‐dose safety study
Source: Epilepsia Open. 2021 May 13;6(3):504–12. doi: 10.1002/epi4.12494 (PMC8408590; doi:10.1002/epi4.12494)
Supplement: Supplementary file 2 — Table S1‐S2 [file EPI4-6-504-s002.docx]

**Supplementary Table 1. Nasal irritation test results**

| **Time point, patients with data, n (%)** | **Moderate frequency usage**  **(<2 doses/month)**  **(n=69)*** | **High frequency usage**  **(2–5 doses/month)**  **(n=80)*** | **Very high frequency usage**  **(>5 doses/month)**  **(n=9)** |
| --- | --- | --- | --- |
| Baseline |  |  |  |
| Grade 0 | 38 (55.1) | 63 (78.8) | 9 (100.0) |
| Grade 1A | 1 (1.4) | 3 (3.8) | - |
| Day 30 |  |  |  |
| Grade 0 | 39 (56.5) | 64 (80.0) | 8 (88.9) |
| Grade 1A | - | 1 (1.3) | 1 (11.1) |
| Day 90 |  |  |  |
| Grade 0 | 35 (50.7) | 62 (77.5) | 9 (100.0) |
| Grade 1A | - | 2 (2.5) | - |
| Day 150 |  |  |  |
| Grade 0 | 35 (50.7) | 53 (66.3) | 9 (100.0) |
| Grade 1A | - | 1 (1.3) | - |
| Grade 1B | - | 1 (1.3) | - |
| Day 210 |  |  |  |
| Grade 0 | 31 (44.9) | 55 (68.8) | 8 (88.9) |
| Day 270 |  |  |  |
| Grade 0 | 26 (37.7) | 50 (62.5) | 8 (88.9) |
| Grade 1A | - | 1 (1.3) | - |
| Day 330 |  |  |  |
| Grade 0 | 27 (39.1) | 40 (50.0) | 6 (66.7) |
| Grade 1A | - | 2 (2.5) | - |
| Grade 1B | - | 1 (1.3) | - |
| Day 365 |  |  |  |
| Grade 0 | 23 (33.3) | 41 (51.3) | 6 (66.7) |
| Grade 1A | - | 3 (3.8) | - |

*Observed instances only; some patients were excluded from the table because the first dose date was after day 30. Grading: 0 no sign of nasal irritation or mucosal erosion, 1A–focal nasal mucosal irritation or inflammation, 1B–superficial mucosal erosion, 2–moderate mucosal erosion, 3–ulceration, and 4–septal perforation.

**Supplementary Table 2. NIH Toolbox Odor Identification Test results**

| **Time point** | **Moderate frequency usage**  **(<2 doses/month)**  **(n=69)*** | **High frequency usage**  **(2–5 doses/month)**  **(n=80)*** | **Very high frequency usage**  **(>5 doses/month)**  **(n=9)** |
| --- | --- | --- | --- |
| Baseline, n | 24 | 35 | 4 |
| Mean (SD) | 1.54 (0.80) | 2.96 (0.79) | 10.55 (3.48) |
| Median (min, max) | 1.35 (1.0, 5.0) | 2.70 (2.0, 5.0) | 10.20 (6.9, 14.9) |
| Day 30, n | 26 | 39 | 4 |
| Mean (SD) | 5.86 (2.56) | 6.21 (2.44) | 7.75 (0.96) |
| Median (min, max) | 6.50 (1.0, 9.0) | 7.00 (1.0, 9.0) | 7.50 (7.0, 9.0) |
| Change from baseline, n | 23 | 34 | 4 |
| Mean (SD) | -0.13 (1.46) | 0.32 (2.09) | 1.50 (1.73) |
| Median (min, max) | 0.0 (-2.0, 3.0) | 0.00 (-6.0, 4.0) | 1.50 (0.0, 3.0) |
| Day 90, n | 25 | 39 | 4 |
| Mean (SD) | 6.48 (2.33) | 6.79 (2.03) | 6.75 (1.26) |
| Median (min, max) | 7.00 (2.0, 9.0) | 7.00 (2.0, 9.0) | 7.00 (5.0, 8.0) |
| Change from baseline, n | 22 | 34 | 4 |
| Mean (SD) | 0.09 (1.48) | 0.26 (1.48) | 0.50 (2.38) |
| Median (min, max) | 0.00 (-4.0, 3.0) | 0.00 (-3.0, 4.0) | 0.50 (-2.0, 3.0) |
| Day 150, n | 21 | 33 | 4 |
| Mean (SD) | 6.33 (2.75) | 6.70 (1.88) | 7.25 (0.96) |
| Median (min, max) | 7.0 (1.0, 9.0) | 7.00 (2.0, 9.0) | 7.50 (6.0, 8.0) |
| Change from baseline, n | 19 | 28 | 4 |
| Mean (SD) | 0.00 (1.29) | 0.14 (1.63) | 1.00 (1.41) |
| Median (min, max) | 0.00 (-3.0, 3.0) | 0.00 (-5.0, 3.0) | 1.50 (-1.0, 2.0) |
| Day 210, n | 18 | 33 | 4 |
| Mean (SD) | 6.28 (2.82) | 6.97 (5.28) | 7.25 (1.71) |
| Median (min, max) | 6.5 (1.0, 9.0) | 7.00 (1.0, 33.0) | 7.50 (5.0, 9.0) |
| Change from baseline, n | 16 | 28 | 4 |
| Mean (SD) | 0.25 (1.57) | 0.89 (5.44) | 1.00 (1.41) |
| Median (min, max) | 0.00 (-3.0, 3.0) | 0.00 (-6.0, 26.0) | 0.50 (0.0, 3.0) |
| Day 270, n | 20 | 31 | 4 |
| Mean (SD) | 6.60 (2.74) | 6.61 (2.12) | 7.25 (1.50) |
| Median (min, max) | 7.50 (1.0, 9.0) | 7.00 (3.0, 9.0) | 7.00 (6.0, 9.0) |
| Change from baseline, n | 18 | 27 | 4 |
| Mean (SD) | 0.11 (1.53) | 0.48 (1.55) | 1.00 (1.83) |
| Median (min, max) | 0.00 (-3.0, 3.0) | 1.00 (-3.0, 4.0) | 1.00 (-1.0, 3.0) |
| Day 330, n | 16 | 22 | 2 |
| Mean (SD) | 6.06 (3.07) | 5.73 (2.41) | 8.00 (1.41) |
| Median (min, max) | 7.00 (1.0, 9.0) | 6.00 (2.0, 9.0) | 8.00 (7.0, 9.0) |
| Change from baseline, n | 14 | 19 | 2 |
| Mean (SD) | -0.14 (1.41) | -0.47 (1.78) | 1.00 (1.41) |
| Median (min, max) | 0.00 (-3.0, -2.0) | 0.00 (-5.0, 4.0) | 1.00 (0.0, 2.0) |
| Day 365, n | 14 | 26 | 3 |
| Mean (SD) | 6.43 (2.77) | 6.12 (2.29) | 8.33 (1.56) |
| Median (min, max) | 7.00 (2.0, 9.0) | 6.50 (2.0, 9.0) | 9.00 (7.0, 9.0) |
| Change from baseline, n | 12 | 23 | 3 |
| Mean (SD) | 0.00 (1.41) | -0.09 (1.91) | 1.33 (2.31) |
| Median (min, max) | 0.00 (-2.0, 3.0) | 0.00 (-5.0, 3.0) | 0.00 (0.0, 4.0) |

*Some patients were excluded from the table because the first dose date was after day 30.

Min, minimum; max, maximum; NIH, National Institutes of Health.
